# Breast cancer screening disparities among urban immigrants: a population-based study in Ontario, Canada

**DOI:** 10.1186/s12889-015-2050-5

**Published:** 2015-07-21

**Authors:** Mandana Vahabi, Aisha Lofters, Matthew Kumar, Richard H. Glazier

**Affiliations:** Faculty of Community Services, Daphne Cockwell School of Nursing, Ryerson University, 350 Victoria Street, Toronto, ON M5B 2K3 Canada; Immigration and Settlement Studies, Ryerson University, Toronto, Canada; Ryerson Centre for Global Health and Health Equity, Toronto, Canada; Centre for Research on Inner City Health, Li Ka Shing Knowledge Institute, St. Michael’s Hospital, Toronto, Canada; Department of Family and Community Medicine, University of Toronto, Toronto, Canada; Department of Family and Community Medicine, St. Michael Hospital, Toronto, Canada; Institute for Clinical Evaluative Sciences, Toronto, Canada; Dalla, Lana School of Public Health, University of Toronto, Toronto, Canada

**Keywords:** Breast cancer, Screening mammography, Immigrants, Primary care patient enrollment models, Internationally trained physicians

## Abstract

**Background:**

Breast cancer is one of the leading cause of mortality and morbidity in Canada. Screening is the most promising approach in identification and treatment of the disease at early stage of its development. Research shows higher rate of breast cancer mortality among ethno-racial immigrant women despite their lower incidence which suggests disparities in mammography screening. This study aimed to compare the prevalence of appropriate mammography screening among immigrant and native borne women and determine predicators of low mammography screening.

**Methods:**

We conducted secondary data analyses on Ontario linked social and health databases to determine the proportion of women who were screened during the two- year period of 2010–2012 among 1.4 million screening-eligible women living in urban centres in Ontario. We used multivariate Poisson regression to adjust for various socio-demographic, health care-related and migration related variables.

**Results:**

64 % of eligible women were appropriately screened. Screening rates were lowest among new and recent immigrants compared to referent group (Canadian-born women and immigrant who arrived before 1985) (Adjusted Rate Ratio (ARR) (0.87, 95 % CI 0.85 –0.88 for new immigrants and 0.90, 95 % CI 0.89–0.91 for recent immigrants. Factors that were associated with lower rates of screening included living in low- income neighborhoods, having a male physician, having internationally- trained physician and not being enrolled in primary care patient enrolment models. Those not enrolled were 22 % less likely to be screened compared to those who were (ARR 0.78, 95 % CI 0.77–0.79).

**Conclusion:**

To enhance immigrant women screening rates efforts should made to increase their access to primary care patient enrolment models and preferably female health professionals. Support should be provided to interventions that address screening barriers like language, acculturation limitations and knowledge deficit. Health professionals need to be educated and take an active role in offering screening guidelines during health encounters.

## Background

Despite extensive progress and effort in treatment, breast cancer remains one of the most life threatening conditions among women worldwide. It is estimated that 1 out of 9 Canadian women will be diagnosed with breast cancer during her lifetime and 1 in 29 will die of it [1]. Screening mammography is the most promising approach in early detection and treatment of the disease. The Canadian Task force on Preventive Health Care [2] recommended that women between the ages of 50–69 undergo screening mammography every 2 years. Although the utilization of screening mammography has increased gradually over the past two decades it has been reported to be suboptimal, particularly among certain groups of women including immigrants [3]. Based on the 2008 Canadian Community Population Health Survey, 57 % of recent immigrants (residing in Canada <10 years) self-identified themselves as non-users, compared with 26 % of Canadian-born women [4]. Research shows a higher prevalence of advanced breast cancer, poorer five-year survival, and higher rates of breast cancer mortality among ethno-racial immigrant women despite their lower incidence of breast cancer !which suggests disparities in mammography screening [5–8]. Socio-economic, cultural, linguistic, structural and systemic barriers have been reported as contributing factors to observed disparities [5, 6, 9–15].

The reported breast cancer screening rates may not accurately reflect the actual disparities in mammography screening as they are often based on self-report which is higher than rates derived from objective data in administrative databases [16–18]. Furthermore, the sampling strategies used in community health surveys do not specifically target immigrant ethno-racial groups leading often to small sample sizes which curtails adequate assessment. Hence, the higher prevalence of advanced breast cancer, inadequate participation and representation of ethno-racial immigrant population in Canadian national surveys and reliance on self-report data warrant further investigation of breast cancer screening utilization and its determinants in Canada which has an increasingly diverse immigrant population. In 2011, Canada included over 200 different ethnic groups which contributed to approximately 21 % of its total population and more than half of them lived in Ontario [19].

The objective of this paper is two-fold: first, to compare the prevalence of appropriate breast cancer screening (screening mammography every 2 years) among women living in Ontario by their immigration status; and second to explore the association between appropriate breast cancer screening among immigrant women in Ontario and several individual and structural factors, These factors include age, neighborhood income, length of stay in Canada, co-morbidities, primary care physician visits, periodic health exams, physician’s gender and training, and type of primary care patient enrollment model (PEM). Several types of PEMs co-exist in Ontario. These include a range of different physician reimbursement models and inter-professional teams with incentives for screening and management of chronic conditions.

## Methods

### Study design

We conducted secondary data analyses on multiple linked social and health databases to determine mammography screening prevalence and its determinants among Ontario women by their immigration status.

### Data sources

Data for this study was based on the following linked databases: The Citizenship and Immigration Canada (CIC) database, Ontario Cancer Registry (OCR); Ontario Breast Screening Program (OBSP); Registered Persons Database (RPDB); Ontario Physicians’ Claims Database – OHIP Claims; Institute for Clinical Evaluative Sciences (ICES) Physician Database (IPDB); Canadian Institute for Health Information Discharge Abstract Database (CIHI-DAD), The Client Agency Program Enrolment (CAPE) tables; the OHIP Corporate Provider Database (CPDB), and 2006 Canadian Census. For more information regarding the individual databases, please see Table 1.

**Table 1:** Description of databases used in study

Database	Description
Citizenship and Immigration Canada (CIC)	CIC includes demographic information about individuals’ at their entry into Ontario as permanent residents from 1985 to 2010. It excludes temporary residents (e.g. students, foreign workers and refugee claimants, those immigrants who landed after 2010, those who declared to move to another province but instead moved to Ontario, and those who could not be probabilistically linked too other databases.
Ontario Cancer Registry (OCR)	OCR captures cancer incidence and mortality information of Ontario residents.
Ontario Breast Screening Program (OBSP)	The OBSP is a program of Cancer Care Ontario that provides breast cancer screening for women aged 50–74 years. OBSP database is an Integrated Client Management System database that contains administrative, clinical and demographic data for each client screened in the OBSP.
Registered Persons Database (RPDB)	Includes residential and demographic information of all Ontario’s residents who are eligible for health care coverage. The eligibility includes being Canadian Citizens, landed immigrants or refugees; their primary and permanent residence is in Ontario; and physically reside in Ontario in any 12-month period for a minimum of least 153 days. For those born outside Ontario the health care coverage starts 3 months after their residency begins.
Ontario Physicians’ Claims Database – OHIP claims	Includes billing and diagnostic information submitted by approximately 95 % of Ontario’s physicians.
ICES Physician Database (IPDB)	Comprises information from the Ontario.
	Health Insurance Plan (OHIP) about the health care providers including: demographics (training, year of graduation), specialization, and workload (type of work, place of work, location, payment plan, FTEs).
Canadian Institute for Health Information Discharge Abstract Database (CIHI-DAD)	Includes acute in-patient hospital discharge data (i.e. demographic, administrative and clinical information).
The Client Agency Program Enrolment (CAPE) tables	This is a repository of the association of a registered person with a specific physician at a specific agency in a formally recognized program, including primary care Patient Enrolment Models.
OHIP Corporate Provider Database (CPDB)	This is a provider registry which includes providers’ demographics and their organizations’ characteristics. It also includes providers’ credentials from the College of Physicians and Surgeons of Ontario (CPSO).
2006 Canadian Census	The census provides demographic and statistical data for all people living in Canada.

Information about Ontario’s population eligible for health services and women breast cancer screening was retrieved through a comprehensive research agreement with Ontario’s Ministry of Health and Long term Care. We removed all personal identifiers from the analytic dataset except for year of birth, date of registration with the health insurance plan, and area of residence, and a scrambled unique identifier. The research protocol was approved by Research Ethics Boards at University of Toronto and Sunnybrook Health Sciences Centre in Toronto.

### Study population

Our study cohort was based on the RPDB and included: all women who were alive and continuously eligible for health coverage from April 1, 2010-March 31, 2012; had their most recent postal code in an Ontario Census Metropolitan Area (CMA) which is a geographic area with an average population of approximately 100,000; and were 50–69 years old for the entire study period. The defined age group includes those who were eligible for screening. The study was limited to CMAs to ensure comparison to an appropriate group with similar access to health care and since the majority of Ontario’s population (82 %) lives in CMAs [20]. The 2 year study period corresponds to the recommended time frame stipulated in the provincial guidelines for breast screening. A total of ***1,457,136*** women fit these inclusion criteria.

We grouped women according to their immigration status in the following three mutually exclusive categories: the first group included ***Identified immigrants*** who were captured in CIC data (those who arrived between 1985 and 2010). The second group included women who were not captured in CIC but had registered with the province’s universal health plan after available data begins (April 1, 1993), referred to as ***Recent registrants (RR)***. Although an unknown but small proportion of Canadian-born inter-provincial migrants was included in this group it does include many of the immigrant women who *were not captured in CIC data* i.e. those who immigrated before 1985 and those who immigrated to other provinces before moving to Ontario. The third group included all other women in the study cohort i.e. Canadian-born women and long-term immigrants who arrived before 1985, referred to as ***Long-term residents (LTR)***.

### Outcome measures

To determine screening status for breast cancer, a woman was considered appropriately screened ***if she had received at least one mammogram in the two year period April 1, 2010-March 31, 2012.*** Since mammography can be performed for both screening and diagnostic purposes, we excluded women where the index of suspicion for diagnostic tests was high, i.e. those with any breast cancer before or on April 1, 2010, any mastectomy, lumpectomy, axillary lymph nodes removal, prophylactic ovary removal before or on March 31, 2012.

A total of 50,076 women were excluded because of these conditions. Hence the final cohort consisted of ***1,407,060*** women (738,240 were 50–59 years and 668,820 were 60–69 years). These age groups were used for stratification as there are often differences in screening rates of younger compared to older women.

### Statistical analysis

Baseline characteristics were compared for the three groups of women (i.e. women included in CIC data (identified immigrants), RR, LTR), and all Ontario eligible women, for each age group (i.e. 50–59, 60–69 and overall 50–69 years). Screening rates for identified immigrants captured in CIC were further analyzed by their length of residence in Canada: ***New immigrants*** (i.e. ≤5 years in Canada) ***recent immigrants*** (6–10 years) and ***established immigrants*** (11 years and more). We also conducted univariable and bivariable analyses. For binary and categorical variables, we used Chi-Square Test of association. For continuous variables, we used a 1-way Analysis of Variance (ANOVA) to compare means.

We conducted multivariable analyses using a binary outcome i.e. whether the woman had a mammography in the prescribed two-year period. We used Poisson regression for this analysis because the outcome is common and not rare [21] and the follow-up time is the same for everyone (i.e. mammography every 2 years). Under such circumstances logistic regression would not provide an accurate approximation of adjusted rate ratios (ARR) [21]. Poisson regression was used to provide the adjusted rate ratios (ARR) for the association between recommended screening mammography practices and selected personal and structural variables across the two age groups in our cohort, as well as for the overall cohort. Those variables included neighborhood income, resource utilization (expected health care costs), level of co-morbidities, having a periodic health exam, number of primary care physician visits, type of primary care enrollment model (PEM), gender and Canadian versus international family doctor training. These variables were selected *a priori* based on literature review [22–40]. We used the Generalized Estimating Equations (GEE) analytic approach to Poisson regression to account for the clustering effects of patients nested within a common physician. We used an exchangeable correlation structure to model this. All statistical tests were performed at the 5 % level of significance, two-sided, using SAS 9.3 (SAS Institute, Cary, NC) for UNIX to fit all models and conduct all descriptive analysis (SAS Institute, Cary, NC).

## Results

In our study cohort, 13 % of women were identified immigrants, 5 % recent registrants and the majority (82 %) were long-term residents. Tables 2 and 3 show overall (50–69) and across the two age groups (50–59 vs. 60–69) baseline characteristics of participants. Identified immigrants had the ***highest:*** proportion of women who resided in the lowest two neighborhood income quintiles, periodic health exam, enrollment in Family Health Groups/Comprehensive Care primary care model, solo physicians (not enrolled in a primary care model), and use of internationally-trained physicians compared to the long term residents (LTR) and recent registrants (RR). This pattern was similar across both age groups.

**Table 2:** Baseline characteristics of the 1,407,060 women in the study population who lived in Ontario’s Metropolitan areas for the study period April 1, 2010-March 31, 2012

Variable	Value	Identified Immigrants (CIC)	Long-Term Residents (LTR)	Recent Registrants (RR)	Total	*P*-value
		*N* = 183,332	*N* = 1,160,050	*N* = 63,678	*N* = 1,407,060	
Age	Mean ± SD	58.47 ± 4.96	59.73 ± 5.06	58.58 ± 4.97	59.51 ± 5.06	<.001
Neighbourhood Income Quintile	1	46,786 (25.5 %)	185,528 (16.0 %)	12,224 (19.2 %)	244,538 (17.4 %)	<.001
	2	42,476 (23.2 %)	217,448 (18.7 %)	11,942 (18.8 %)	271,866 (19.3 %)	
	3	38,008 (20.7 %)	227,020 (19.6 %)	12,218 (19.2 %)	277,246 (19.7 %)	
	4	32,983 (18.0 %)	250,884 (21.6 %)	13,196 (20.7 %)	297,063 (21.1 %)	
	5	22,722 (12.4 %)	274,939 (23.7 %)	13,808 (21.7 %)	311,469 (22.1 %)	
	Missing	357 (0.2 %)	4,231 (0.4 %)	290 (0.5 %)	4,878 (0.3 %)	
Rural	Missing	54 (0.0 %)	584 (0.1 %)	28 (0.0 %)	666 (0.0 %)	<.001
	N	180,538 (98.5 %)	979,001 (84.4 %)	56,293 (88.4 %)	1,215,832 (86.4 %)	
	Y	2,740 (1.5 %)	180,465 (15.6 %)	7,357 (11.6 %)	190,562 (13.5 %)	
RUBs^a^	0–1	14,983 (8.2 %)	81,301 (7.0 %)	7,932 (12.5 %)	104,216 (7.4 %)	<.001
	2	19,297 (10.5 %)	135,999 (11.7 %)	7,517 (11.8 %)	162,813 (11.6 %)	
	3	113,006 (61.6 %)	682,675 (58.8 %)	35,691 (56.0 %)	831,372 (59.1 %)	
	4+	36,046 (19.7 %)	260,075 (22.4 %)	12,538 (19.7 %)	308,659 (21.9 %)	
ADG	Mean ± SD	6.07 ± 3.54	6.04 ± 3.57	5.63 ± 3.73	6.02 ± 3.58	<.001
ADG^b^	0	10,992 (6.0 %)	46,210 (4.0 %)	6,158 (9.7 %)	63,360 (4.5 %)	<.001
	1–5	73,144 (39.9 %)	509,832 (43.9 %)	26,407 (41.5 %)	609,383 (43.3 %)	
	6–9	68,070 (37.1 %)	408,761 (35.2 %)	21,265 (33.4 %)	498,096 (35.4 %)	
	10+	31,126 (17.0 %)	195,247 (16.8 %)	9,848 (15.5 %)	236,221 (16.8 %)	
Periodic health exam	No	77,957 (42.5 %)	545,245 (47.0 %)	31,544 (49.5 %)	654,746 (46.5 %)	<.001
	Yes	105,375 (57.5 %)	614,805 (53.0 %)	32,134 (50.5 %)	752,314 (53.5 %)	
GP visits	Mean ± SD	5.29 ± 5.09	4.32 ± 4.99	4.16 ± 5.02	4.44 ± 5.01	<.001
Physician enrollment model	FHG/CCM	103,187 (56.3 %)	365,975 (31.5 %)	24,968 (39.2 %)	494,130 (35.1 %)	<.001
	FHN	107 (0.1 %)	7,986 (0.7 %)	175 (0.3 %)	8,268 (0.6 %)	
	FHO	40,064 (21.9 %)	375,618 (32.4 %)	17,047 (26.8 %)	432,729 (30.8 %)	
	FHT	12,468 (6.8 %)	267,771 (23.1 %)	9,891 (15.5 %)	290,130 (20.6 %)	
	No-Care	7,814 (4.3 %)	40,832 (3.5 %)	5,361 (8.4 %)	54,007 (3.8 %)	
	No-Grp	18,722 (10.2 %)	72,567 (6.3 %)	5,047 (7.9 %)	96,336 (6.8 %)	
	Other	970 (0.5 %)	29,301 (2.5 %)	1,189 (1.9 %)	31,460 (2.2 %)	
Physician sex	Missing	8,361 (4.6 %)	49,423 (4.3 %)	5,951 (9.3 %)	63,735 (4.5 %)	<.001
	F	66,562 (36.3 %)	442,882 (38.2 %)	25,021 (39.3 %)	534,465 (38.0 %)	
	M	108,409 (59.1 %)	667,745 (57.6 %)	32,706 (51.4 %)	808,860 (57.5 %)	
Physician training	Domestic	92,969 (50.7 %)	927,987 (80.0 %)	41,210 (64.7 %)	1,062,166 (75.5 %)	<.001
	International	81,672 (44.5 %)	180,827 (15.6 %)	16,310 (25.6 %)	278,809 (19.8 %)	
	Missing	8,691 (4.7 %)	51,236 (4.4 %)	6,158 (9.7 %)	66,085 (4.7 %)	

^a^RUB = Resource Utilization Bands are part of the Johns Hopkins Adjusted Clinical Group® (ACG®) Case Mix System. The RUBs are used to categorize patients based on their expected use of health care resources and range from 0 (lowest expected health care costs) to 5 (highest expected health care costs)

^b^ADG = Aggregated Diagnosis Groups are part of the Johns Hopkins Adjusted Clinical Group® (ACG®) case-mix system. The ADGs are used to measure the level of co-morbidity and range from 0 (no diagnosis group) to 32 (32 distinct diagnosis groups)

**Table 3:** Baseline characteristics of the 1,407,060 women in the study population who lived in Ontario’s metropolitan areas for the study period April 1, 2010-March 31, 2012 by age group

	Women aged 50–59 on April 1, 2010				Women aged 60–69 on April 1, 2010			
	(*n* = 738,240)				(*n* = 688,820)			
Value	Identified Immigrants (CIC)	Long-Term Residents (LTR)	Recent Registrants (RR)	*P*-Value	Identified Immigrants (CIC)	Long-Term Residents (LTR)	Recent Registrants (RR)	*P*-Value
Sociodemographic factors								
N	113,234	586,325	38681		70,098	573,725	24,997	
Mean age (SD)	55.09 ± 2.25	55.41 ± 2.29	55.13 ± 2.25	<.001	63.92 ± 2.83	64.14 ± 2.81	63.93 ± 2.81	<.001
Neighborhood income quintile, number (%)								
1	28,275 (25.0 %)	91,910 (15.7 %)	7,360 (19.0 %)	<.001	18,511 (26.4 %)	93,618 (16.3 %)	4,864 (19.5 %)	<.001
2	26,111 (23.1 %)	108,645 (18.5 %)	7,242 (18.7 %)		16,365 (23.3 %)	108,803 (19.0 %)	4,700 (18.8 %)	
3	23,477 (20.7 %)	115,145 (19.6 %)	7,369 (19.1 %)		14,531 (20.7 %)	111,875 (19.5 %)	4,849 (19.4 %)	
4	20,729 (18.3 %)	128,266 (21.9 %)	8,021 (20.7 %)		12,254 (17.5 %)	122,618 (21.4 %)	5,175 (20.7 %)	
5 (highest)	14,417 (12.7 %)	140,152 (23.9 %)	8,515 (22.0 %)		8,305 (11.8 %)	134,787 (23.5 %)	5,293 (21.2 %)	
Missing	225 (0.2 %)	2,207 (0.4 %)	174 (0.4 %)		132 (0.2 %)	2,024 (0.4 %)	116 (0.5 %)	
Health care-related factors								
RUBS^a^ (Categories)								
0–1	9,497 (8.4 %)	48,454 (8.3 %)	4,932 (12.8 %)	<.001	5,486 (7.8 %)	32,847 (5.7 %)	3,000 (12.0 %)	<.001
2	12,925 (11.4 %)	78,814 (13.4 %)	5,014 (13.0 %)		6,372 (9.1 %)	57,185 (10.0 %)	2,503 (10.0 %)	
3	70,280 (62.1 %)	345,365 (58.9 %)	21,848 (56.5 %)		42,726 (61.0 %)	337,310 (58.8 %)	13,843 (55.4 %)	
4+	20,532 (18.1 %)	113,692 (19.4 %)	6,887 (17.8 %)		15,514 (22.1 %)	146,383 (25.5 %)	5,651 (22.6 %)	
ADG^b^								
Mean ± SD	5.99 ± 3.48	5.76 ± 3.50	5.49 ± 3.65	<.001	6.19 ± 3.63	6.33 ± 3.62	5.84 ± 3.84	<.001
Median (IQR)	6 (3–8)	5 (3–8)	5 (3–8)	<.001	6 (4–9)	6 (4–9)	6 (3–8)	<.001
ADG (Categories)								
0	6,643 (5.8 %)	26,788 (4.4 %)	3,733 (9.4 %)	<.001	4,401 (6.3 %)	19,600 (3.4 %)	2,456 (9.8 %)	<.001
1–5	46,562 (40.3 %)	277,514 (46.1 %)	16,922 (42.8 %)		27,045 (38.6 %)	236,864 (41.3 %)	9,695 (38.8 %)	
6–9	43,307 (37.5 %)	206,960 (34.4 %)	13,058 (33.0 %)		25,690 (36.6 %)	208,421 (36.3 %)	8,549 (34.2 %)	
10+	18,985 (16.4 %)	90,955 (15.1 %)	5,802 (14.7 %)		12,962 (18.5 %)	108,840 (19.0 %)	4,297 (17.2 %)	
Periodic health exam)								
Yes	66,273 (58.5 %)	309,149 (52.7 %)	19,684 (50.9 %)		39,102 (55.8 %)	305,656 (53.3 %)	12,450 (49.8 %)	<.001
GP visits								
Mean ± SD	5.07 ± 4.99	4.09 ± 5.14	4.08 ± 5.08	<.001	5.65 ± 5.22	4.54 ± 4.82	4.28 ± 4.93	<.001
Median (IQR)	4 (2–7)	3 (1–5)	3 (1–6)	<.001	5 (2–8)	3 (2–6)	3 (1–6)	<.001
Enrollment model								
FHG/CCM	62,820 (55.5 %)	186,247 (31.8 %)	15,594 (40.3 %)	<.001	40,367 (57.6 %)	179,728 (31.3 %)	9,374 (37.5 %)	<.001
FHN	64 (0.1 %)	4,027 (0.7 %)	94 (0.2 %)		43 (0.1 %)	3,959 (0.7 %)	81 (0.3 %)	
FHO	25,369 (22.4 %)	188,473 (32.1 %)	10,143 (26.2 %)		14,695 (21.0 %)	187,145 (32.6 %)	6,904 (27.6 %)	
FHT	8,136 (7.2 %)	134,474 (22.9 %)	5,809 (15.0 %)		4,332 (6.2 %)	133,297 (23.2 %)	4,082 (16.3 %)	
No-Care	4,757 (4.2 %)	21,964 (3.7 %)	3,186 (8.2 %)		3,057 (4.4 %)	18,868 (3.3 %)	2,175 (8.7 %)	
No-Group	11,510 (10.2 %)	36,318 (6.2 %)	3,160 (8.2 %)		7,212 (10.3 %)	36,249 (6.3 %)	1,887 (7.5 %)	
Other	578 (0.5 %)	14,822 (2.5 %)	695 (1.8 %)		392 (0.6 %)	14,479 (2.5 %)	494 (2.0 %)	
Physician sex								
Female	42,281 (37.3 %)	233,124 (39.8 %)	15,633 (40.4 %)		24,281 (34.6 %)	209,758 (36.6 %)	9,388 (37.6 %)	<.001
Male	65,832 (58.1 %)	326,954 (55.8 %)	19,504 (50.4 %)		42,577 (60.7 %)	340,791 (59.4 %)	13,202 (52.8 %)	
Physician training								
Domestic	59,139 (52.2 %)	469,827 (80.1 %)	24,896 (64.4 %)	<.001	33,830 (48.3 %)	458,160 (79.9 %)	16,314 (65.3 %)	<.001
International	48,779 (43.1 %)	89,374 (15.2 %)	10,118 (26.2 %)		32,893 (46.9 %)	91,453 (15.9 %)	6,192 (24.8 %)	

^a^RUB = Resource Utilization Bands are part of the Johns Hopkins Adjusted Clinical Group® (ACG®) Case Mix System. The RUBs are used to categorize patients based on their expected use of health care resources and range from 0 (lowest expected health care costs) to 5 (highest expected health care costs)

^b^ADG = Aggregated Diagnosis Groups are part of the Johns Hopkins Adjusted Clinical Group® (ACG®) case-mix system. The ADGs are used to measure the level of co-morbidity and range from 0 (no diagnosis group) to 32 (32 distinct diagnosis groups)

### Screening rates

Tables 4 and 5 display overall and across the age groups screening rates by immigration groups and other socio-demographic and health-related characteristics. Sixty-four percent of women 50–69 were appropriately screened. Overall, screening rates were lower for identified immigrants (57 %) and RR (57 %) compared to 66 % among LTR (Table 4). There were higher appropriate screening rates among older women (67 %) compared to younger women (62 %) (Table 5). Furthermore, breast cancer screening rates increased with increasing neighborhood income, greater utilization of health services, and higher number of co-morbidities, having a periodic health exam, having a primary care physician, patient enrolment models particularly Family Health Teams (FHT), Family Health Organizations (FHO) and Family Health Networks (FHN), having a female physician, and having a Canadian-trained physician (Tables 4 and 5). FHO and FHN are blended capitation models and FHT is a team-based model with physicians reimbursed through blended capitation or blended salary.

**Table 4:** Breast cancer screening rates for Ontario women aged 50 – 69 who lived in Metropolitan areas for the study period April 1, 2010-March 31, 2012

Variable	Value	CIC	LTR	RR	Total
*N*		57 %	66 %	57 %	64 %
Income quintile	1	54 %	59 %	50 %	58 %
	2	57 %	64 %	55 %	62 %
	3	58 %	66 %	57 %	65 %
	4	60 %	68 %	60 %	67 %
	5	60 %	70 %	62 %	69 %
	Missing	62 %	59 %	51 %	59 %
Rural	No	57 %	66 %	57 %	64 %
	Yes	57 %	65 %	56 %	65 %
	Missing	54 %	66 %	57 %	64 %
RUBS (Categorized)	1	12 %	30 %	13 %	26 %
	2	45 %	58 %	48 %	56 %
	3	62 %	70 %	65 %	69 %
	4+	66 %	70 %	66 %	69 %
ADG (Categorized)	0	4 %	15 %	5 %	12 %
	1–5	51 %	62 %	54 %	60 %
	6–9	67 %	74 %	69 %	72 %
	10+	70 %	73 %	70 %	72 %
General physical check-up	No	36 %	49 %	37 %	47 %
	Yes	72 %	81 %	77 %	79 %
Enrollment model	FHG/CCM	59 %	66 %	60 %	65 %
	FHN	62 %	67 %	62 %	67 %
	FHO	62 %	69 %	64 %	68 %
	FHT	65 %	71 %	65 %	70 %
	No-Care	11 %	23 %	12 %	20 %
	No-Grp	49 %	55 %	47 %	53 %
	Other	66 %	67 %	59 %	67 %
Physician sex	Female	64 %	72 %	66 %	71 %
	Male	56 %	64 %	57 %	63 %
	Missing	13 %	30 %	17 %	26 %
Physician training	Domestic	61 %	68 %	62 %	67 %
	International	57 %	65 %	58 %	62 %
	Missing	15 %	31 %	18 %	28 %

**Table 5:** Breast cancer screening rates for Ontario Women who lived in metropolitan areas for the study period April 1, 2010-March 31, 2012, by age group

Screening rates: ages 50–59						Screening rates: ages 60–69			
Variable	Value	CIC	LTR	RR	Total	CIC	LTR	RR	Total
Overall		58.4 %	63.2 %	56.0 %	62.1 %	55.0 %	68.8 %	57.9 %	66.9 %
Income quintile	1	54.3 %	56.0 %	49.1 %	55.2 %	52.4 %	62.1 %	51.2 %	60.1 %
	2	58.1 %	60.8 %	53.7 %	59.9 %	54.5 %	67.1 %	56.2 %	65.1 %
	3	59.3 %	63.3 %	56.0 %	62.3 %	54.9 %	69.0 %	57.8 %	67.1 %
	4	61.1 %	65.6 %	59.4 %	64.7 %	57.0 %	71.0 %	60.5 %	69.4 %
	5	61.6 %	67.8 %	61.0 %	66.9 %	58.6 %	72.6 %	63.1 %	71.5 %
	Missing	62.7 %	55.7 %	44.8 %	55.6 %	62.1 %	62.8 %	59.5 %	62.6 %
Rural	No	58.5 %	63.6 %	56.4 %	62.3 %	54.9 %	68.8 %	57.7 %	66.7 %
	Yes	55.7 %	61.3 %	52.9 %	60.9 %	59.3 %	68.8 %	59.4 %	68.4 %
	Missing	56.1 %	65.7 %	57.1 %	63.9 %	46.2 %	65.8 %	<=5	64.9 %
RUBS (Categorized)	1	13.8 %	30.6 %	13.7 %	26.7 %	7.7 %	30.2 %	10.9 %	25.8 %
	2	46.6 %	56.1 %	48.3 %	54.4 %	42.6 %	60.0 %	47.9 %	57.8 %
	3	63.8 %	68.2 %	64.7 %	67.3 %	59.9 %	72.8 %	66.0 %	71.1 %
	4+	68.1 %	66.9 %	64.1 %	67.0 %	63.3 %	71.8 %	67.4 %	70.8 %
ADG (Categorized)	0	4.4 %	15.2 %	5.2 %	12.3 %	2.4 %	15.5 %	5.1 %	12.3 %
	01-May	52.0 %	59.6 %	54.1 %	58.3 %	48.8 %	64.4 %	54.8 %	62.5 %
	06-Sep	68.0 %	71.4 %	67.7 %	70.7 %	64.0 %	75.6 %	69.9 %	74.2 %
	10+	71.9 %	70.4 %	69.0 %	70.6 %	67.7 %	75.0 %	71.2 %	74.1 %
General physical check-up	No	37.0 %	45.7 %	35.2 %	43.9 %	35.4 %	52.9 %	38.8 %	50.5 %
	Yes	73.6 %	78.9 %	76.1 %	77.9 %	70.5 %	82.8 %	77.2 %	81.2 %
Enrollment model	FHG/CCM	60.6 %	64.0 %	59.9 %	63.0 %	56.8 %	68.9 %	60.7 %	66.4 %
	FHN	70.3 %	64.0 %	62.8 %	64.1 %	48.8 %	70.7 %	61.7 %	70.3 %
	FHO	63.1 %	66.1 %	62.9 %	65.6 %	60.9 %	71.6 %	65.2 %	70.7 %
	FHT	65.4 %	67.9 %	62.9 %	67.5 %	63.2 %	73.7 %	67.2 %	73.2 %
	No Care	11.8 %	21.1 %	12.2 %	18.7 %	8.6 %	24.5 %	12.9 %	21.4 %
	No Model	50.3 %	52.0 %	45.7 %	51.2 %	46.7 %	57.5 %	48.1 %	55.4 %
	Other	65.9 %	64.0 %	57.3 %	63.8 %	66.6 %	70.7 %	61.3 %	70.3 %
Physician sex	Female	65.3 %	70.1 %	65.1 %	69.1 %	62.2 %	75.2 %	67.4 %	73.6 %
	Male	57.4 %	61.2 %	55.9 %	60.4 %	54.2 %	67.4 %	58.5 %	65.6 %
	Missing	14.5 %	27.7 %	16.1 %	24.6 %	11.1 %	32.4 %	17.4 %	28.8 %
Physician training	Domestic	62.1 %	65.3 %	60.6 %	64.8 %	59.8 %	70.8 %	63.8 %	69.9 %
	International	58.5 %	62.5 %	58.5 %	60.9 %	54.3 %	67.6 %	58.1 %	63.8 %
	Missing	16.5 %	29.0 %	17.8 %	26.0 %	12.9 %	34.1 %	18.9 %	30.4 %

**Fig. 1:**
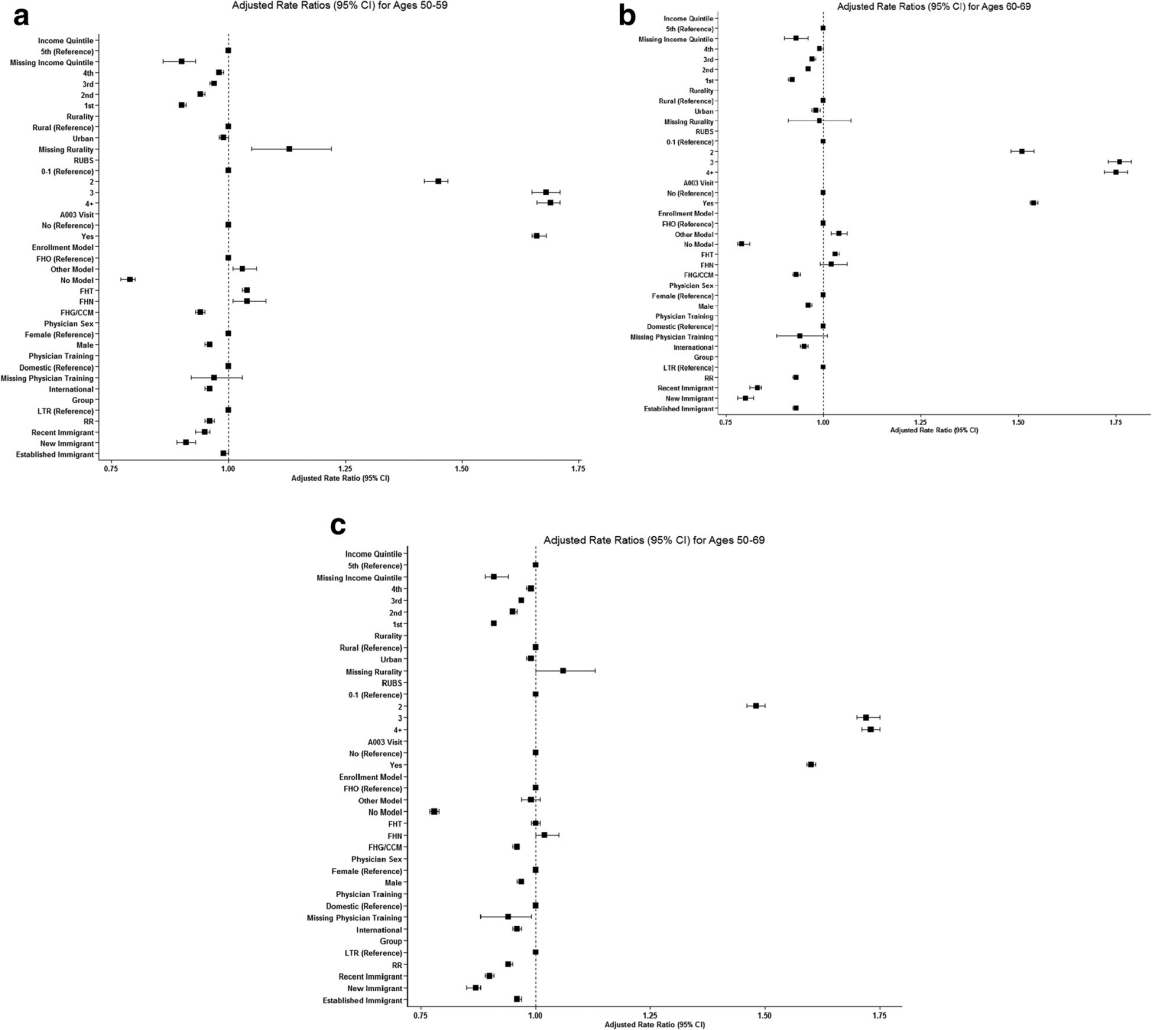
**a** Screening Rates and Adjusted Rate Ratios for 50–59 Age Group. **b** Screening Rates and Adjusted Rate Ratios for 60–69 Age Group. **c** Screening Rates and Adjusted Rate Ratios for 50–69 Age Group

In our multivariate analyses, we found significant differences in appropriate screening by immigrant group. The screening rate for ***new immigrants*** (i.e. ≤5 years in Canada) was 50 % as opposed to 52 % for ***recent immigrants*** (6–10 years) and 60 % for ***established immigrants*** (11 years and more) (data not shown). The older new immigrants had the lowest screening rates (46 %) while the younger established immigrants had the highest (60 %) (data not shown). Table 6 and Fig. 1a, b, c show ARRs for appropriate screening rates for overall participants and by age group and socio-demographic, immigration and health related characteristics. Immigrants had significantly lower rates compared to LTR (ARR 0.87, 95 % CI 0.85–0.88) for new immigrants, (ARR 0.90, 95 % CI 0.89–0.91) for recent immigrants, (ARR 0.96, 95 % CI 0.96–0.97) for established immigrants), and this was most pronounced among the older age group. The income gradient was similar for both age groups, with women living in the lowest income neighborhoods being approximately 10 % less likely to be appropriately screened than their high income counterparts (ARR 0.91, 95 % CI 0.91–0.91). Screening rates increased with increasing health care use. Patient enrolment models were associated with an increased likelihood of being screened; those not enrolled were 22 % less likely to be screened compared to those who were enrolled in FHO (ARR 0.78, 95 % CI 0.77–0.79). Having a periodic health exam increased the likelihood of being appropriately screened by 60 % (ARR 1.60, 95 % CI 1.59–1.61).

**Table 6:** Adjusted rate ratios (ARR) and [95 % confidence intervals] for appropriate breast cancer screening for women, from multivariable Poisson regression, by age group

Variable	Effect	Ages 50–59	Ages 60–69	Ages 50–69
Income quintile	1	0.90 [0.90 - 0.91]	0.92 [0.91 - 0.92]	0.91 [0.91 - 0.91]
	2	0.94 [0.94 - 0.95	0.96 [0.96 - 0.96]	0.95 [0.95 - 0.96]
	3	0.97 [0.96 - 0.97]	0.97 [0.97 - 0.98]	0.97 [0.97 - 0.97]
	4	0.98 [0.98 - 0.99]	0.99 [0.99 - 1.00]	0.99 [0.98 - 0.99]
	Missing	0.90 [0.86 - 0.93]	0.93 [0.90 - 0.96]	0.91 [0.89 - 0.94]
	5 (Reference)			
Rural	Missing	1.13 [1.05 - 1.22]	0.99 [0.91 - 1.07]	1.06 [1.00 - 1.13]
	Urban	0.99 [0.98 - 1.00]	0.98 [0.97 - 0.99]	0.99 [0.98 - 0.99]
	Rural (Reference)			
RUBS	2	1.45 [1.42 - 1.47]	1.51 [1.48 - 1.54]	1.48 [1.46 - 1.50]
	3	1.68 [1.65 - 1.71]	1.76 [1.73 - 1.79]	1.72 [1.70 - 1.75]
	4+	1.69 [1.66 - 1.71]	1.75 [1.72 - 1.78]	1.73 [1.71 - 1.75]
	0-1 (Reference)			
General physical check-up	Yes	1.66 [1.65 - 1.68]	1.54 [1.53 - 1.55]	1.60 [1.59 - 1.61]
	No (Reference)			
Enrollment model	FHG/CCM	0.94 [0.93 - 0.95]	0.93 [0.92 - 0.94]	0.96 [0.95 - 0.96]
	FHN	1.04 [1.01 - 1.08]	1.02 [0.99 - 1.06]	1.02 [1.00 - 1.05]
	FHT	1.04 [1.03 - 1.04]	1.03 [1.03 - 1.04]	1.00 [0.99 - 1.01]
	No Model	0.79 [0.77 - 0.80]	0.79 [0.78 - 0.81]	0.78 [0.77 - 0.79]
	Other Model	1.03 [1.01 - 1.06]	1.04 [1.02 - 1.06]	0.99 [0.97 - 1.01]
	FHO (Reference)			
Physician sex	Male	0.96 [0.95 - 0.96]	0.96 [0.96 - 0.97]	0.97 [0.96 - 0.97]
	Female (Reference)			
Physician training	International	0.96 [0.95 - 0.96]	0.95 [0.94 - 0.96]	0.96 [0.95 - 0.97]
	Missing	0.97 [0.92 - 1.03]	0.94 [0.88 - 1.01]	0.94 [0.88 - 0.99]
	Domestic (Reference)			
Group	Established Immigrant	0.99 [0.99 - 1.00]	0.93 [0.92 - 0.93]	0.96 [0.96 - 0.97]
	New Immigrant	0.91 [0.89 - 0.93]	0.80 [0.78 - 0.82]	0.87 [0.85 - 0.88]
	Recent Immigrant	0.95 [0.93 - 0.96]	0.83 [0.81 - 0.84]	0.90 [0.89 - 0.91]
	RR	0.96 [0.95 - 0.97]	0.93 [0.92 - 0.93]	0.94 [0.94 - 0.95]
	LTR (Reference)			
GP visits		1.00 [1.00 - 1.00]	1.00 [1.00 - 1.00]	1.00 [1.00 - 1.00]

### Discussion

Considering the national target participation rate of 70 %, our study showed that overall breast cancer screening rates in Ontario are still suboptimal with less than two-thirds of eligible women being appropriately screened. However, rates of appropriate screening were significantly lower among immigrant women than for long term residents and varied by their length of stay in Canada. Older immigrant women aged 60–69 years had significantly lower rates than younger immigrant women aged 50–59 years. Higher rates of screening were associated with being enrolled in a primary care PEM, having higher use of health services and co-morbidities, and having a periodic health exam. Factors that were associated with lower rates of screening included living in low- income neighborhoods, having a male physician, and having an internationally-trained physician.

Our finding of lower rates of screening mammography among immigrant women supports the screening disparities reported in other studies [4–6, 13–16] but also shows an increase in screening use with increasing length of residence in Canada. The effect of acculturation particularly among those living in Canada more than 10 years seem to be playing a role in promoting screening practices. Cultural beliefs and values (such as culturally-based fatalistic beliefs, viewing cancer as taboo and stigmatic, belief that cancer can be caused just by thinking or talking about it) in conjunction with factors such as linguistic and economic challenges after migration, and limited knowledge of available health care services in the host country are common barriers to access and use of screening services by immigrant women [5, 9, 10, 22–28]. The notion of prevention and early detection has been reported to be quite alien to non-European immigrants, particularly those from developing countries. The emphasis on treatment of symptoms and anomalies in the home countries may hinder immigrant women to seek medical care while asymptomatic post migration [5, 10, 27–30]. These insights can be used to guide the design and implementation of culturally and linguistically appropriate breast cancer screening education and programs to address their pre-existing beliefs about breast cancer and screening.

Prior studies have reported that immigrants, particularly newcomers, are disproportionately included in low income neighborhoods [15, 30, 31]. People with low incomes may have competing priorities and not be able to make time for preventive care. Lobb and colleagues [32] identified several factors that could sway immigrants’ access to screening services in those neighborhoods, like fear of losing wages since screening services are offered during working hours, belief that payment was required for the tests, transportation, and need for interpretation.

The role of primary care providers in encouraging preventive services has been highlighted in several studies [33–36]. We found that frequent contact with the health care system and having physical check-ups significantly increased screening. Having more contact with the health care system provides ample opportunities for healthcare providers to educate and inform women about the benefits of screening. We also found that lower rates of screening were associated with having internationally- trained physicians. Physicians’ inadequate knowledge of screening guidelines and lack of recommendations have been identified as key deterrents in screening participation and reinforcement of misperception that some groups are not at risk for cancer [31–33]. Internationally- trained physicians may have a lack of awareness and familiarity with Canadian screening guidelines or may be contending with the imprints of previous training and practice which may have focused heavily on treatment of disease. Similar to results from other studies [37, 38], physician characteristics such as being female were associated with higher rates of screening. Differences in beliefs and practices of male and female physicians regarding the effectiveness, referral and follow up of screening, as well as patient preference for a female provider have been identified to contribute to the higher rates of mammography screening [37, 38]. Finally, the type of physician enrollment model was associated with use of screening. As expected and shown in prior studies [39, 40], rates were higher for those PEMs (e.g. FHO, FHTs and FHNs) where there are financial incentives to provide preventive care, and lowest for physicians not enrolled in any model. This may suggests that patients’ enrollment in PEMs should be encouraged in order to promote regular and timely uptake of screening by women in general and immigrants in particular.

Our study had several limitations. First, the use of administrative data limits the ability to address causation or account for some confounders such as religion and ethnicity that may affect women’s participation in screening. Second, those immigrant women who were not captured through CIC, but arrived between 1985 and 1993, would be included in the referent group. Moreover, the referent group also included immigrant women who arrived before 1985. However, the proportion of women this applies to, although unknown, should be relatively small and our results are likely not greatly affected. According to Canadian Census 2006, about 75 % of migrants to Ontario were from other countries and the remainder was from other Canadian provinces, some of whom were also immigrants [41]. If we had been able to isolate Canadian-born women, we then would expect to observe a larger disparity than reported here. Third, we used neighborhood income which is an ecological variable, as a proxy for women’s income, which may lead to ecological fallacy. However, the ecological-level variables are commonly used in the health equity studies and can provide conservative estimates of socioeconomic effects [42]. Finally, we used Poisson regression which is more commonly used for count variables as the dependent variable. This was done because our outcome (whether the woman had a breast cancer screening in the past 2 years) is quite common, so logistic regression, which gives odds ratios, would not give accurate estimates of rate ratios. It would give incorrectly large magnitudes for ratios. In cases where the outcome is common, Poisson regression can be used to calculate rate ratios as long as the follow-up time is the same for everyone, which in our case it was (i.e. in the past 2 years) [21, 40]. This approach will give a conservative confidence interval since Poisson errors are over-estimates of binominal errors, however because of our large sample size our confidence intervals remained relatively narrow. Despite these limitations, our study had many strengths. It is a large, population-based study with broad inclusion criteria which contained all women age 50–69 with health coverage in Ontario’s urban areas. The use of objective data instead of self-report is another advantage which overcomes biases inherent in self-report. We also stratified all the analyses by age group to determine if there were differences based on women’s age. Furthermore, the study looked at multiple individual and system related variables which permit us to identify high risk groups and tailor interventions and strategies for promoting breast cancer screening rates in underserved populations.

## Conclusion

The results from this study suggest that a multi-pronged approach may be required to increase screening rates in Ontario and narrow the gap between immigrants, particularly recent, and longer-term residents. For instance, education may be beneficial for primary care providers, particularly internationally-trained physicians, about the importance of encouraging women to have screening. This education could particularly target those providers who are not in patient enrollment models and foreign trained physicians. This may possibly be coupled with empowering women by educating them about the health services that are available and the importance of breast screening. Given the finding that having a physician is a gateway to receiving screening which has also been corroborated in other studies, existing strategies in Ontario such as the Health Care Connect program that aims to attach unattached individuals to primary care physicians can be leveraged to ensure that all women who require one, should have a primary care provider. A targeted approach might be taken to reach specific groups such as newcomers, ethnic groups, and those living in certain geographic areas. Education should include culturally and linguistically appropriate breast cancer screening information (e.g., positive framing and non-fear provoking messaging, use of third person) and non-medical information like location, cost and hours of service. It may be beneficial to explore policies and strategies targeted at ensuring accessibility of screening program by vulnerable underserved populations (e.g. offering services after work hours, patient transportation subsidies, out-reach screening programs). For instance, system level interventions like structured recall system based on electronic medical records (EMR) from physicians’ offices, reminder letters from organized breast cancer screening programs, and use of patient outreach and navigation models [43], may have some potential for promoting breast cancer screening among immigrants and could further be explored in helping to reduce/close this kind of equity gap. Future research is required on 1) attempting to address causation by causal modelling analysis and 2) conducting cost-benefit analysis of our recommendations and prioritizing them for policy makers’ decisions.
